# The cellular landscape by cryo soft X-ray tomography

**DOI:** 10.1007/s12551-019-00567-6

**Published:** 2019-07-04

**Authors:** J. Groen, J. J. Conesa, R. Valcárcel, E. Pereiro

**Affiliations:** 1 Mistral Beamline, Alba Light Source (Cells), Cerdanyola del Valles, 08290 Barcelona, Spain; 2 Department of Macromolecular Structures, Cantoblanco, 28049 Madrid, Spain

**Keywords:** X-ray microscopy, Cryo soft X-ray tomography, Correlative microscopy, Organelle atlas

## Abstract

Imaging techniques in structural cell biology are indispensable to understand cell organization and machinery. In this frame, cryo soft X-ray tomography (cryo-SXT), a synchrotron-based imaging technique, is used to analyze the ultrastructure of intact, cryo-preserved cells at nanometric spatial resolution bridging electron microscopy and visible light fluorescence. With their unique interaction with matter and high penetration depth, X-rays are a very useful and complementary source to obtain both high-resolution and quantitative information. In this review, we are elaborating a typical cryo correlative workflow at the Mistral Beamline at the Alba Synchrotron (Spain) with the goal of providing a cartographic description of the cell by cryo-SXT that illustrates the possibilities this technique brings for specific localization of cellular features, organelle organization, and particular events in specific structural cell biology research.

## Introduction

Structural biology has been one of the greatest beneficiaries from the development of advanced microscopic techniques. Among the two most common are transmission electron microscopy (TEM) and visible light microscopy (VLM). While VLM has been around for centuries, it was always restricted to the diffraction limit of light. Only due to recent advances are we able to surpass that limit. New methods, like single-molecule localization microscopy (SMLM) or the development of new components, like for example the spinning disk or the airy scan unit for the confocal microscope, in combination with elaborate data processing, allow us to localize the fluorescence signal with a resolution down to 30 nm. One feature of SMLM, which can be considered a disadvantage, is that the location of the signal requires the use of fluorescent dyes. These give excellent specificity, but only few can be used at the same time and therefore the non-labeled molecules or structures, which might contain extra information, are invisible.

While VLM itself is a very old technique, its ability to visualize highly resolved structures is very recent. TEM has been around for several decades and has a higher resolving power. With a resolution below 5 nm, it has been a classical technique to visualize cell structures, but the nature of electrons imposes some restrictions. First, multiple scattering limits the thickness of the sample that can be imaged. Second, resin embedding and staining are required and therefore the cell structure is not anymore the native one. Due to the thickness limitation, it is very challenging to obtain a full three-dimensional (3D) view of the cell. In order to reach a resolution of 5 nm or lower, the sample has to be sectioned into very thin sections of about a 100 nm. Apart from possible artifacts caused by the sectioning, each section has to be imaged individually and chronologically, which is very time-consuming. Recent advances, like focused ion beam/ scanning electron microscopy (FIB-SEM) or serial block-face imaging, have greatly improved the automated acquisition of 3D information, making it much easier and faster. Cryo-capabilities have also been developed so that cryo-FIB milling of the sample allows now for cryo electron tomography (ET) avoiding staining and dehydration, but cryo-ET is still a non-routine technique compared with the classical TEM on epoxy resin–embedded sections.

**Fig. 1:**
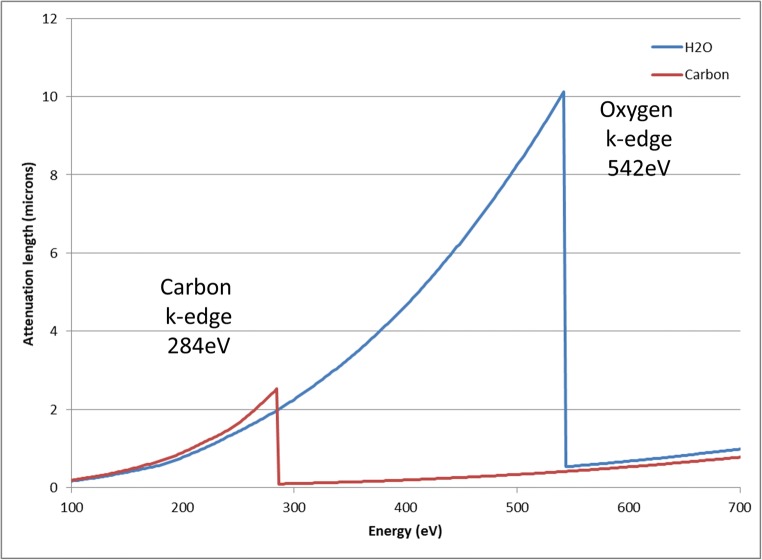
Attenuation coefficients of carbon and water. The area between the carbon K edge and oxygen K edge is known as the water window

Both VLM and TEM can provide useful data to answer a variety of biological questions; however, the gap of spatial resolution achievable between them and the volume analyzed with each technique is big and difficult to handle when trying to combine information, leaving therefore many questions unanswered. Cryo soft X-ray tomography (cryo-SXT) can for a large part fill this gap and can be considered the bridge between these two techniques. Although very new, it is a powerful technique to study biological samples (Carrascosa et al. 2009; Chiappi et el. 2016 ; Chichón et al. 2012; Chen et al. 2016; Duke et al. 2014; Hagen et al. 2012; Hanssen et al. 2011, 2012; Hummel et al. 2012; Jacobsen 1999; Le Gros et al. 2016 ; Parkinson et al. 2008 ; Pérez-Berná et al. 2016; Schneider et al. 2010; Uchida et al. 2009; Varsano et al. 2016). Unlike conventional TEM, it does not need any staining reagents to obtain sufficient contrast. This is possible due to the unique interaction of X-rays with different elements. The Mistral beamline (Alba Synchrotron) is dedicated to cryo-SXT and spectroscopy applications (Pereiro et al. 2009) (Sorrentino et al. 2015). Soft X-rays are especially useful for imaging cellular structures since, depending on the energy chosen, it is possible to visualize different elements that have an absorption edge within this spectrum. Most commonly used is the water window (Fig. 1), which is located between the carbon K edge (284 eV) and the oxygen K edge (543 eV). At 520 eV carbon-rich elements (like cellular membranes) absorb strongly, while oxygen-rich elements (like water/cytoplasm), absorb poorly. This is directly dependent on the carbon and nitrogen content of the absorbing structure, meaning that the contrast is in a direct relationship with the elemental composition (following the Beer-Lambert law). Because of this, SXT allows quantitative studies as the transmission intensity through the sample follows:

1 $$ {I}_z={I}_0\times {e}^{-\mu \rho z} $$

where *μ* is the mass absorption coefficient (cm^2^/g) of the cell structures, *ρ* is their density (g/cm^3^), *z* is their thickness, and *I*_0_ is the incoming flux. A tomography is therefore the 3D reconstruction of the linear coefficient *μ*_l_ (cm^−1^) = *μ* × *ρ* of the sample being imaged. Due to this, the signal in each voxel is representative for its content giving quantitative information and allowing for statistical analysis. Based on their absorption coefficient, organelles that appear structurally the same can be differentiated. Using this same principle, the signal can also be utilized for quantification of structures, for instance. Different elements can be imaged and quantified using their absorption edges within the water window. By imaging just below and just above the L-edges of calcium, Gal et al. (2018) visualized and quantified calcium (absorption edge 353 eV) in algae in the complete cellular context to understand Ca-containing organelles in calcifying and non-calcifying species. With some adjustments to the preparation protocol, it is also possible to visualize absorption edges that are outside the water window. Conesa et al. (2016) have visualized and quantified the concentration of iron oxide superparamagnetic nanoparticles (absorption edge 709 eV), within the whole cellular context.

Apart from all the advantages and features mentioned above, the greatest advantage of cryo-SXT is its ability to image unstained, whole frozen cells up to 10-μm thick and with a resolution down to 30 nm (Schneider et al. 2010) in a volume of few microns (Chichón et al. 2012). While this resolution is significantly lower than EM, in some cases, it can be an advantage to image at a somewhat lower resolution but with a much larger volume. Especially when features in the context of the whole cell are to be studied, like overall organelle organization or structural changes due to a viral infection (Pérez-Berná et al. 2016).

Cryo-SXT is an imaging technique that can easily be used to complement techniques such as visible light fluorescence microscopy (VLFM) or TEM. Correlative workflows including the first one are usually necessary to combine specific structure or molecule localization within the cellular context. Using correlative cryo-VLFM and cryo-SXT (cryo-CLXM), specific organelles such as autophagosomes have been identified by Duke et al. (2014). More recently, super-resolution CLXM has been gaining interest. This can be done before freezing on fixed cells to study the early cholesterol crystal formation in macrophages (Varsano et al. 2016, 2018) or after freezing them (Spink et al. 2018). A full cryogenic approach has indeed higher efficiency as the motion of molecules in the time lapse between imaging techniques and vitrification are avoided, as well as possible detachment of the interesting cells during vitrification. Moreover, the natural cellular contrast available at the water window energy range is maintained since no chemical fixation agents (mainly composed of carbon) are needed for VLFM imaging.

Cryo hard X-ray fluorescence microscopy (XRF) can also be combined with cryo-SXT. Cryo-XRF allows detecting specific elements with very high sensitivity (down to ppb). Therefore, combining both techniques on the same cell will allow detecting specific elements within the cellular context with the possibility of quantification (Kapishnikov et al. 2017a, b).

The combination of cryo-SXT and TEM information can also give additional insights into the processes. For the moment, this has been done non-correlatively to visualize specific features at higher magnification on resin-embedded thin sections. But one could also imagine exploring a correlative approach in which the same cell could first be imaged by cryo-SXT, and then the region of interest could be imaged by cryo-ET after producing a cryo-FIB lamella of it.

In this review, we want to highlight what is the information that can be achieved by cryo-SXT. With this aim, we will give a short introduction to the microscope setup first, as well as a detailed step by step description of the typical workflow employed at the Mistral beamline. We will then focus our attention to provide an atlas description of the cell organelles with the aim to complement the one made by Müller et al. (2012), to finally discuss some of the observable features.

## Beamline characteristics and workflow

**Fig. 2:**
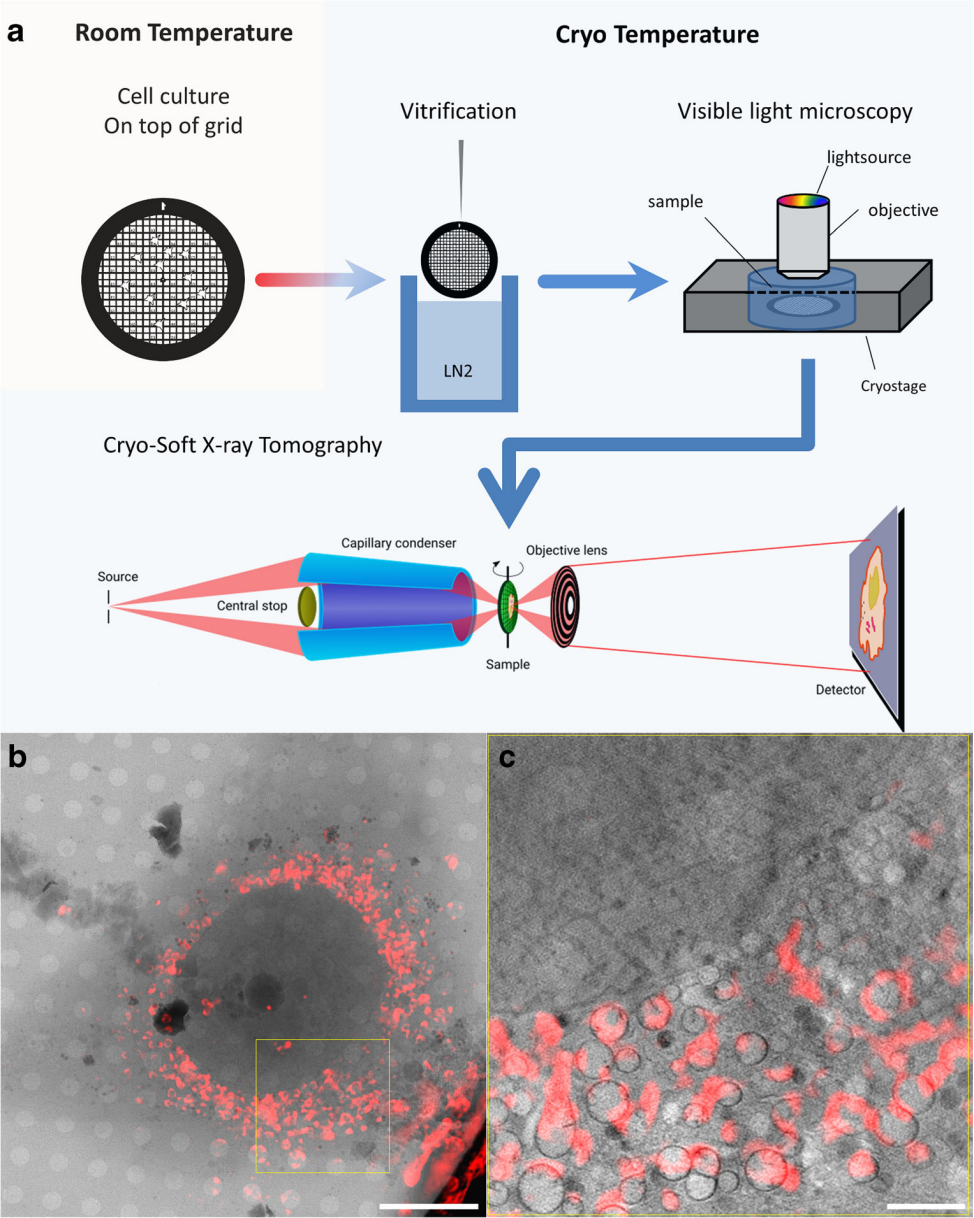
**a** A typical workflow at the Mistral Beamline. Cells are grown on top of TEM grids and vitrified. Once frozen, the grids are checked with visible light. Using a regular fluorescence microscope setup and a cryo-stage, the quality of the sample (cell density, flatness of the grid, vitrification success) is checked and potential areas of interest are selected. If available, high-resolution fluorescence data can be collected as well. After fluorescence imaging, the samples are transferred to the TXM chamber. An on-line fluorescence microscope is used to re-locate the areas previously imaged, and tilt-series are collected from the previously imaged cells. **b** A mosaic image overlaid with the fluorescent signal coming from a mitochondrial stain obtained by cryo structured illumination microscopy (SIM). **c** One slice of the reconstructed volume from the yellow box in **b**. Most of the signal is coming from the mitochondrial membranes, which is the target of the dye. Scale bar: B, 10 μm; C, 2 μm

Prior to the acquisition of cryo-SXT data, some preparative steps have to be performed. Figure 2 a shows a schematic of the workflow including these preparative steps: growing the cells on the grids (or depositing them on top when cells in suspension are used), vitrification, and finally screening the samples by cryo visible light microscopy. The only step not included in this scheme is the processing of the data.

Cells are seeded on top of Quantifoil (R2/2, Quantifoil Micro Tools)-coated Au-TEM finder grids (G200F1, Gilder) and left to settle and attach for (at least) 24 h. Before freezing, fluorescent dyes are added for later identification of the cell region of interest positions on the grid. This is followed by the addition of a small aliquot of gold fiducial markers for later alignment of the angular projections prior to reconstruction. The fiducial markers are allowed to attach to the surface for 30 s, before the excess liquid is removed by blotting with a Whatman #1 filter paper to leave a thin layer of liquid covering the cells. Immediately after blotting, the grid is plunged into liquid ethane, kept at ~ − 170 °C. The vitrified grids are then first checked by visible light microscopy using a Linkam cryo-stage that maintains the grids in cryo-conditions, to select the better-preserved samples with the right confluency and ice thickness. Cryo-epifluorescence is usually used to identify and localize promising areas for cryo-SXT later, but also for correlative purposes (see Fig. 2b, c). The best samples are then transferred to the transmission X-ray microscope (TXM) chamber and, using an on-line fluorescence visible light microscope, the previously identified areas are again easily located. In this work, the data shown is from either primary mouse fibroblasts or NIH-3T3 fibroblast-like cells and we used MitoTracker (MitoTracker™ Red CMXRos, ThermoFisher) as a fluorescent dye.

The X-rays, coming from a bending magnet, are focused and monochromatized to a 10–15 × 10–15-μm^2^ field of view at the sample position. This is done with a series of mirrors that focus the X-rays to produce a small secondary source which will feed the TXM with light. A monochromator is used to select the photon energy required for the experiment. The energy can be changed during the experiment to, for example, look at the absorption edge of a different element. From the secondary source, the beam diverges to fill a glass capillary which acts as a condenser lens that focuses the light onto the sample. The sample stage is fully motorized to allow X, Y, and Z motions as well as rotation. The transmitted light through the sample is focalized by the objective lens, a diffractive Fresnel Zone plate (FZP) positioned at a specific focal length which depends on the energy used. The diffracted light coming out from the FZP is projected onto a thermoelectrically cooled back-illuminated CCD camera forming a magnified image of the sample. There are usually two FZP lenses available, a 40-nm outermost zone width and a 25 nm. The objective lens sets the resolution achievable and the depth of field.

**Fig. 3:**
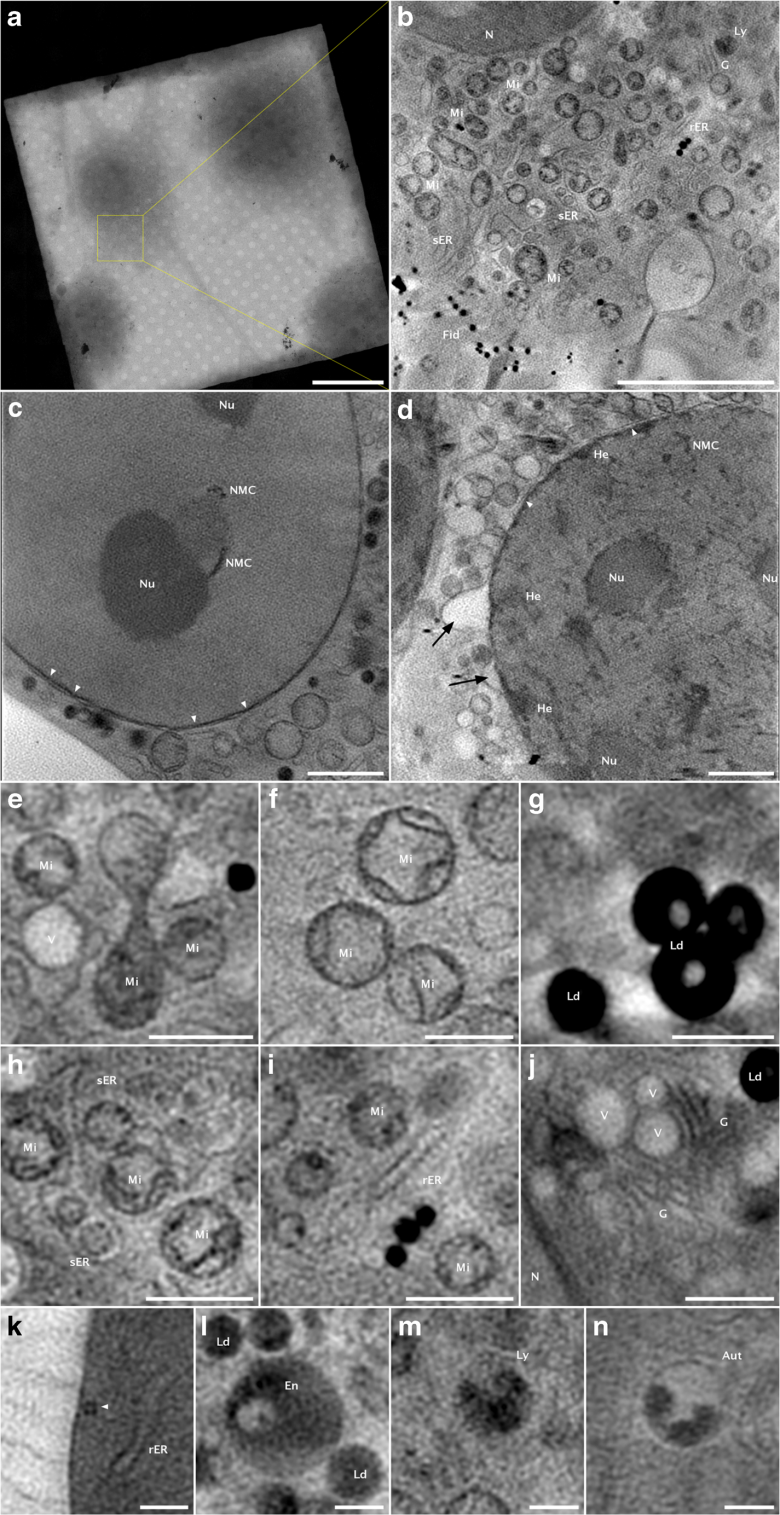
An overview of cryo soft X-ray data of mammalian cells (fibroblast and NIH-3T3 cells). The different structures have been labeled as follows: N, nucleus; NMC, nuclear membrane channel; Nu, nucleolus; Mi, mitochondria; He, heterochromatin; G, Golgi apparatus; rER, rough endoplasmic reticulum; sER, smooth endoplasmic reticulum; Fid, fiducial markers; Ld, lipid droplets; V, vesicles; Ly, lysosome; En, endosome; Aut, autophagosome. **a** A mosaic overview of a square within the grid with a cell of interest. Within the mosaic, areas for acquiring tilt-series are selected. The yellow box represents the field of view of the camera. **b** One slice of the reconstructed volume from the yellow box shown in **a**. **c, d** One slice of a reconstructed volume showing the N of two different cells. In **c**, two Nu are visible, as well as some NMC. The white arrows aim at pores within the double nuclear membrane. In **d**, Nu is visible and He structures can be seen close to the nuclear membrane. The white arrowheads point to the double membrane. The black arrows point to a detachment of the outer layer of the nuclear membrane, which is called nuclear blebbing. **e,f** Two Mi forms we were able to observe. A small and elongated form (e) and some swollen Mi (**f**). **g** The two forms of Ld usually appear as filled or with an empty core. **h, i** ER can be found between other organelles. While rER is elongated and usually easy to find, sER membranes are thinner and appear to be randomly distributed. **j** The G is usually surrounded by low absorbing V and appears as parallel oriented elongated structures close to the N. **k**, **l**, **m**, **n** Endocytic vesicles in different stages. Depending of the developmental and metabolic state of the cell, different forms can be found. Scale bars: **a** 20 μm; **b** 5 μ; **c**, **d** 2 μm; **e**, **f**, **g**, **h**, **i**, **j** 1 μm; **k**, **l**, **m**, **n** 0.5 μm

Once the grids have been loaded into the TXM (4 samples at a time), a first grid is chosen and an overview of the different grid squares on which interesting positions have been previously localized by cryo-epifluorescence are imaged to find the specific cells (see Figs. 2b and 3a). Within this so-called mosaic map, areas are selected for the tomographic acquisition using the epifluorescence information. The FZP used to image the cells shown in this publication has a 40-nm outermost zone width, which allows the sample to rotate 141° (− 70° to + 70°). Once a suitable location has been found, 141 transmission projections of a field of view of 10–15 × 10–15 μm^2^ are acquired, at every angle between − 70° and + 70° in steps of 1°. In order to obtain a 3D tomographic reconstruction of the cell, a number of processing steps are required. First, the individual projections are normalized by a flat field image which is the incoming flux *I*_0_ from Eq. 1. This normalization also takes into account the (possibly different) exposure time, as well as the slight decrease of the electron beam current during the acquisition. A wiener deconvolution taking into account the experimental impulse response of the optical system (Otón et al. 2016) is then applied to the normalized data in order to increase the image quality (point spread function deconvolution), and finally, the Naperian logarithm is taken to reconstruct the linear absorption coefficient. The resulting stacks are then loaded into IMOD software (Kremer et al. 1996), and, using the fiducial markers, the individual projections are aligned to the common tilt-axis. Tomo3D (Agulleiro and Fernandez 2011) is then used to reconstruct the aligned stacks using the Simultaneous Iterative Reconstruction Technique (SIRT) algorithm.

When it comes to reconstruction algorithms, several are available. Due to the sample only being able to rotate up to −/+ 70 degrees, there is 20 degrees on both sides missing to cover the full angular range. This missing information is called the missing wedge, and one of the biggest challenges in algorithm development is to compensate it. Weighted back projection (WBP) (reviewed in Radermacher 2006) was the first algorithm used for reconstructions. However, other algorithms such as SIRT (Gilbert 1972) and algebraic reconstruction techniques (ART) (Gordon et al. 1970) are performing better. Recent algorithms are mostly trying to overcome the missing wedge, and the artifacts it induces, like for example model-based iterative reconstruction (MBIR) (Yan et al. 2019).

## Organelle atlas

Extensive TEM libraries coming from epoxy resin thin sections of different cell-lines and their organelles are available. Concurrently, Müller et al. (2012) started an atlas to show the most prominent features and also compare them to epoxy resin TEM images. Since the structural appearance of organelles from cryo-SXT data and classical TEM can differ considerably due to the different sample preparation (hydrated vs. dehydrated), we aim at complementing the cryo-SXT organelle atlas showing structures already described but also some that were not yet shown. Figure 3 a shows a mosaic map in which four cells are clearly visible. The yellow squared area points out to the chosen area for collecting the tomogram shown in Fig. 3b. Figure 3 b shows one slice of the reconstructed volume containing most of the organelles we discuss in this section.

In Fig. 3 c and d, the nucleus of a fibroblast (3C) or a fibroblast-like (3D) cell (NIH-3T3) are shown, respectively. Each of them is presenting different features. In Fig. 3c, the nucleolus, two nuclear channels, and the double nuclear membrane with nuclear membrane pores are easily identifiable. In Fig. 3d, the double nuclear membrane presents blebbing at several points. Another structural feature that can clearly be seen in 3D is the localization of heterochromatin which shows up as darker regions within the nucleus as the DNA is more tightly packed compared with euchromatin.

In Fig. 3 e and f, we show two examples of mitochondria. In Fig. 3e, we see two small round mitochondria and one elongated. In both, the cristae are visible, although very thin. Usual sizes found in fibroblasts range from 0.4 to 0.8 μm in diameter. In Fig. 3f, the mitochondria shown are swollen and their sizes are between 0.8 and 1 μm in diameter; however, in NIH-3T3 cells, we have found that the swelling could increase their diameter to up to 1.4 μm. Previous studies have shown that this mitochondrial swelling is triggered by the increased presence of TGF-β (Negmadjanov et al. 2015). This cytokine is released in fibroblast and NIH-3T3 cells upon differentiation, and the mitochondrial swelling is an answer to the increased demand for energy.

Figure 3 g shows lipid droplets in the two forms we are able to observe them. In some cases, we found them to have a lighter core, while in other cases we found them to be completely filled. In both cases, their absorption coefficient is very high due to their high carbon (lipid) content. Their high absorption together with their size makes them one of the easiest organelles to find and recognize, even in the angular projections prior to reconstruction.

In Fig. 3 h–j, some of the finer membrane structures are depicted. Fig. 3 h shows smooth endoplasmic reticulum (sER), Fig. 3 i shows the rough endoplasmic reticulum (rER), and Fig. 3 j shows the Golgi apparatus. These organelles are difficult to visualize because their membrane size is close to the resolution limit of the cryo-SXT (Mitra et al. 2004). sER can typically be recognized by their thin-walled, almost randomly distributed membranes that sometimes contain vesicle-like structures. The rER is easier to find due to their apparently thicker membranes. Although the resolution is not high enough to visualize ribosomes, their presence on the rER might be the reason that it appears to be more absorbing. The Golgi apparatus can look very similar to rER. It usually appears as multiple layers of elongated membranes. It can typically be found close to the nucleus and surrounded by low-density vesicles.

In Fig. 3k–n, several endocytic vesicles are shown. Fig. 3 k shows the process of endocytosis in action where a small vesicle is being formed. The other three show some forms of endocytic compartments. Based on their morphology alone, they are most likely an endosome (3L), a lysosome (3M), and an autophagosome (3N). However, their exact classification depends also on, for example, protein populations within. Endosomes are a precursor for many endocytic vesicles or multivesicular bodies. Because of this, they can have different appearances and the one shown here is just one example. Although their exact classification can be challenging, they can be recognized by their usually high absorbing content with one or multiple low absorbing vesicles within. Lysosomes can be recognized by their thin outer membrane and internal granular structures. The cup-shaped high absorbing form shown in Fig. 3 n is typical of autophagosomes. Although this is the easiest to recognize, it is only one of its possible forms.

## Discussion

One of the advantages of cryo-SXT is the penetration depth and the unique interaction with matter. Although having less spatial resolution than TEM, cryo-SXT has the ability to image the whole cell unstained, allowing for statistical analysis of organelles, for instance, or the visualization of cellular reorganization or modification due to specific processes. With an exposure time of 1 s, a tilt-series of 141 projection images can be acquired in less than 3 min (141 s for imaging plus motor movements) which allows for high throughput data collection. Considering also the sample preparation, cryo-SXT does not require dehydration, embedding in resin, and, as previously mentioned, staining. This not only reduces preparation time, it also allows to use the acquired signal quantitatively to determine densities of cell components, but also densities of other compounds containing elements with an absorption edge within the soft X-ray spectrum. Another advantage is that it is a non-destructive technique and can be combined with other imaging techniques, such as for example VLM and X-ray fluorescence.

Such as for ET, the missing wedge information produces a reconstructed volume with a non-isotropic spatial resolution and some features, depending on their orientation with respect to the axis of rotation, will not be resolved. Two approximations can be used to reduce this phenomenon. The first one aims to decrease the missing wedge by completing the missing information by means of compensating algorithms (MBIR, for instance) (Venkatakrishnan et al. 2013; Yan et al. 2019). Another approximation is to perform dual tilt tomography (Mastronarde 1997) which consists of the acquisition of two tomograms in the same area with the ninety-degree difference in the tilt-axis position. In this way, the missing wedge becomes a missing pyramid decreasing the artifacts associated with the missing of information. This approximation has been implemented at the Mistral beamline (Valcárcel et al. 2018). In cryo-SXT, the resolution and the depth of field (DoF) are defined by the objective lens used. The 40-nm FZP has a theoretical DoF of 2.6 μm, which in practice means that the spatial resolution of the full volume will not be isotropic as mammalian cells are generally thicker (cytoplasm is about 3–5 μm and the nucleus can be up to 10 μm) and images will be hampered by the out of focus information. However, this issue can be tackled by new data acquisition schemes that allow for an extension of the depth of field of the lens (Otón et al. 2017).

As already elaborated in the introduction, cryo-SXT is a valuable tool to unravel ultrastructural features while at the same time providing statistically relevant data. In addition, in combination with other microscopic techniques, it can provide information that exceeds the possibilities of either technique alone. With an increasing interest in cryo visible light fluorescence, and the accompanying development of new technologies, it will become easier to develop new correlative workflows between the two techniques at similar resolution. The combination of cryo-SXT and TEM can also provide very useful additional information. Because for TEM the sample needs to be thinned before imaging, it is often hard to find a specific event within the 3D cellular context. A new correlative approach that could be explored would be to image such an event within the full 3D cellular landscape by cryo-SXT first and then to visualize that precise event at higher resolution with TEM or cryo-ET by milling the sample to a lamella that would contain the event as the 3D coordinates would be known. And since nowadays, the first step of sample preparation for high-resolution TEM is usually cryo-fixation, the subsequent steps can easily be applied after cryo-SXT imaging, although attention will be required on the total dose given to the sample, since it can affect the integrity of the cells and the grid itself (Chichón et al. 2012).

Indeed, the use of cryo-SXT in combination with other techniques is mandatory to understand such a complex system as a cell. And every cell type is unique and can therefore show differences in morphology or absorption. Because of this, it is important to create an atlas with data on a wide range of cells such as the one started by Müller and colleagues (Müller et al. 2012) that we have tried here to complement adding new structures.
